# Modeling Microtubule Counterion Distributions and Conductivity Using the Poisson-Boltzmann Equation

**DOI:** 10.3389/fmolb.2021.650757

**Published:** 2021-03-25

**Authors:** Boden B. Eakins, Sahil D. Patel, Aarat P. Kalra, Vahid Rezania, Karthik Shankar, Jack A. Tuszynski

**Affiliations:** ^1^Department of Electrical and Computer Engineering, University of Alberta, Edmonton, AB, Canada; ^2^Department of Electrical and Computer Engineering, University of California, Santa Barbara, Santa Barbara, CA, United States; ^3^Department of Chemistry, Princeton University, Princeton, NJ, United States; ^4^Department of Physical Sciences, MacEwan University, Edmonton, AB, Canada; ^5^Department of Physics, University of Alberta, Edmonton, AB, Canada; ^6^Department of Mechanical and Aerospace Engineering, Politecnico di Torino, Turin, Italy; ^7^Department of Oncology, University of Alberta, Edmonton, AB, Canada

**Keywords:** cytoskeleton, microtubules, counter-ions, conductivity, bio-electricity, Poisson-Boltzmann, COMSOL

## Abstract

Microtubules are highly negatively charged proteins which have been shown to behave as bio-nanowires capable of conducting ionic currents. The electrical characteristics of microtubules are highly complicated and have been the subject of previous work; however, the impact of the ionic concentration of the buffer solution on microtubule electrical properties has often been overlooked. In this work we use the non-linear Poisson Boltzmann equation, modified to account for a variable permittivity and a Stern Layer, to calculate counterion concentration profiles as a function of the ionic concentration of the buffer. We find that for low-concentration buffers ([KCl] from 10 μ*M* to 10 *mM*) the counterion concentration is largely independent of the buffer's ionic concentration, but for physiological-concentration buffers ([KCl] from 100 to 500 *mM*) the counterion concentration varies dramatically with changes in the buffer's ionic concentration. We then calculate the conductivity of microtubule-counterion complexes, which are found to be more conductive than the buffer when the buffer's ionic concentrations is less than ≈100 *mM* and less conductive otherwise. These results demonstrate the importance of accounting for the ionic concentration of the buffer when analyzing microtubule electrical properties both under laboratory and physiological conditions. We conclude by calculating the basic electrical parameters of microtubules over a range of ionic buffer concentrations applicable to nanodevice and medical applications.

## 1. Introduction

**Figure 1:**
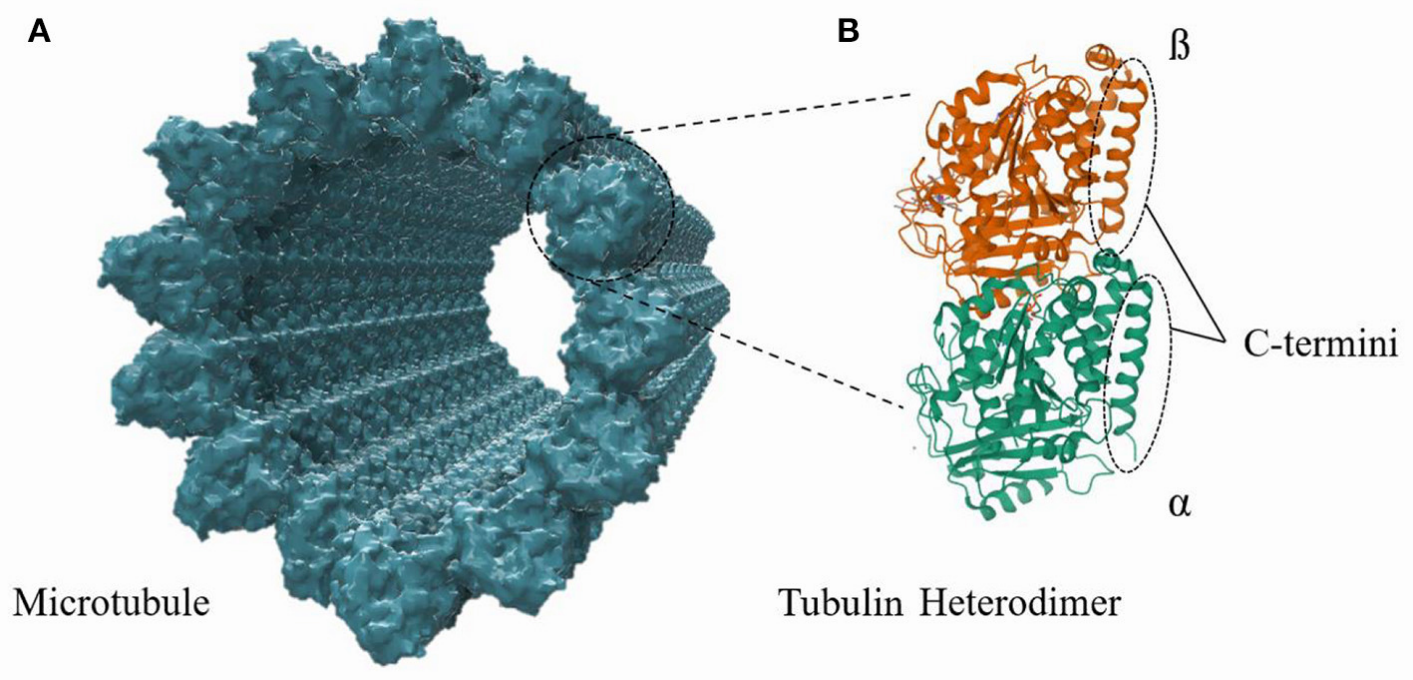
A structural representation of an MT, composed of tubulin heterodimer subunits, is shown in **(A)**. The structure of an individual tubulin heterodimer is shown in **(B)**. This schematic is not to the correct scale and conformation (see Supplementary Material), but the illustration highlights that the c-termini lie on and protrude outward from the outer MT wall.

Microtubules (MTs) are cytoskeletal protein polymers of great interest in fundamental biological research and nanodevice design. A single MT is a relatively stiff (flexural rigidity: ≈ 2.2 · 10^−23^ *Nm*^2^; Gittes et al., 1993), cylindrical polymer with an outer radius of 12.5 nm and a hollow central interior, referred to as the lumen, of radius 8.4 nm. Each MT cylinder is composed of 13 vertical stacks of α, β-tubulin, which are slightly offset from one another to form a helical tubulin lattice. Every tubulin monomer (either α or β) has a ≈4 nm long and ≈1 nm thick C-terminus tail which protrudes from the outer cylinder of the MT; the structures of an MT and a tubulin heterodimer are shown in Figure 1. Tubulin heterodimers are unusually highly charged compared to other proteins, with a total electric charge of ≈−53 *e* per dimer, ≈50% of which resides on the C-termini. While the total tubulin charge, and the fraction of it which lies on the C-termini, varies with the tubulin isotype, there exists a substantial electrostatic attraction of cations in the surrounding buffer to the MT surface. These ions (mainly cations) form a layer of “counterions” on the MT outer surface, acting as charge carriers along the MT length. While there have been numerous experimental and theoretical papers written on the resulting electrical properties of MTs (Priel et al., 2006; Van den Heuvel et al., 2007; Priel and Tuszyński, 2008; Satarić et al., 2009; Sekulić et al., 2011; Sekulić and Satarić, 2012; Santelices et al., 2017; Cantero et al., 2018, 2019; Kalra et al., 2020b; Tuszynski et al., 2020), questions about MT electrical properties remain. In this work, a computational model is developed to calculate the counterionic behavior around a microtubule and predict MT conductivity and other fundamental electrical parameters.

MTs have been shown to demonstrate a number of interesting electrical properties, including long-distance propagation of ionic signals, signal amplification, electrical oscillations, and memristive responses (Priel et al., 2006; Priel and Tuszyński, 2008; Satarić et al., 2009; Sekulić et al., 2011; Sekulić and Satarić, 2012; Cantero et al., 2018, 2019; Tuszynski et al., 2020). These properties have been theorized to play an important role in biological processes, and may be leveraged for the fabrication of MT nanodevices in the near future (Van den Heuvel et al., 2006; Isozaki et al., 2015; Kalra et al., 2020a, 2021). Of these electrical properties, ionic signal propagation is the most well-studied, and the propagation of coherent ionic signals along MTs is a necessary component for memristive behavior and ionic signal amplification (Freedman et al., 2010; Tuszynski et al., 2020). Experimental measurements on isolated MTs are very difficult, and, to our knowledge, signal propagation along an individual MT has only been demonstrated in a single experiment to date (Priel et al., 2006). Theoretical analyses of MTs, however, have been more successful, and a number of authors have analyzed the propagation of ionic signals by modeling MTs as transmission lines. The result of this work is a model in which ionic signals can propagate along MTs as solitons capable of traveling intracellular distances before diminishing below the thermal noise fluctuations (Sekulić and Satarić, 2012).

Transmission line models of MTs are based on theoretical calculations of the conductivity, capacitance, and inductance of short sections of the MT. Positive counterions which have condensed around the MT have a conductance, both axial and radial to the MT surface; an inductance, due to an MT's inherent helical structure; and a capacitance, due to the separation of the positive counterions from the corresponding negative charge (Priel and Tuszyński, 2008; Satarić et al., 2009; Sekulić et al., 2011; Sekulić and Satarić, 2012). Notably, this capacitance has been modeled to arise across a theorized depleted layer, which separates the counterions from bulk solution. This depleted layer is also hypothesized to act as a shield between the counterions and bulk solution (Priel and Tuszyński, 2008; Satarić et al., 2009; Sekulić et al., 2011; Sekulić and Satarić, 2012). Further work has extended calculations of capacitance to account for non-linear effects due to the motion of C-termini, and this is expected to result in stable ionic solitons (Sekulić et al., 2011; Sekulić and Satarić, 2012). However, in all transmission line models to date, calculations of the electrical parameters have (a) assumed the presence of a depleted layer which separates the counterions from bulk solution and (b) analyzed the structure of the counterionic cloud using Manning's theory. This work revisits these approximations in order to arrive at more reliable parameter estimates under a range of ionic concentration conditions.

## 2. Theoretical Models of Counterion Condensation

Manning's theory of counterion condensation has been used extensively to analyze biological polyelectrolytes—such as **f-**actin, MTs, and DNA—in ionic solutions. Importantly, Manning's counterion condensation theory is based on the assumptions that the thickness of the polyelectrolyte is negligible—it is treated as a line charge—and that the surrounding solution is highly dilute (Manning, 1969; Lamm and Pack, 2010). Starting from these postulates, Manning used thermodynamics to show that polyelectrolytes have a critical charge density. If the linear charge density is greater than this threshold, counterions will condense along the polyelectrolyte until the effective charge of the polyelectrolyte and condensed ions equals the critical charge density. Therefore, the effective charge of the polyelectrolyte is equal to the real charge divided by the screening coefficient *S* = λ_*B*_/*b*, where λ_*B*_ is the Bjerrum length, *b* = *L*/*P*, and P is the number of charged groups over a distance L (Manning, 1969). Naturally, modeling a polyelectrolyte (particularly an MT which has a diameter of 25 nm) as a linear charge distribution, and assuming the surrounding solution to be highly dilute, compromises the ability of Manning's model to accurately predict counterionic behavior. To address these drawbacks numerous works have attempted to analyze polyelectrolytes, particularly DNA, using the Poisson-Boltzmann equation instead (Chu et al., 2007; Gruziel et al., 2008; Lamm and Pack, 2010; Kirmizialtin et al., 2012).

Typically, the Poisson-Boltzmann (PB) equation is considered to be a more accurate model of counterionic condensation than Manning's theory. A free-energy analysis by Stigter concluded that Manning's counterion condensation theory was artificially constrained and should be considered an approximation of the more accurate PB theory (Stigter, 1995). Moreover, Manning counterion condensation is predicted by, and has been analyzed under the framework of, the PB equation (Ramanathan, 1983; O'Shaughnessy and Yang, 2005; Lamm and Pack, 2010). These studies suggest that the PB equation would be a better starting point for estimating MT electrical properties.

Another reason to not use Manning's theory when analyzing MTs is that it is only applicable when λ_*D*_ >> *r* where λ_*D*_ is the Debye length and *r* is the radius of the polyelectrolyte (O'Shaughnessy and Yang, 2005). This condition arises from the initial assumption that the polyelectrolyte can be modeled as a linear charge distribution. The radius of an MT is 12.5 nm, indicating that Manning's theory is only applicable for ionic solution concentrations which are much <1 mM (significantly lower than physiological ionic concentrations which are between 150 and 400 mM) (Van Eunen et al., 2010; van Eunen and Bakker, 2014). Sekulić and Satarić (2012) justify the application of Manning's theory to higher ionic concentrations by analyzing individual protofilaments in an MT which have a radius of 2.5 nm. However, analyzing protofilaments individually may not be accurate, as Manning's theory is designed for polyelectrolytes in solution, not joined to other polyelectrolytes. Furthermore, the Debye length under physiological conditions is ≈0.8 nm, so Manning's theory is still outside of its range of applicability when analyzing individual protofilaments. Therefore, the PB equation is likely a more accurate computational tool to analyze the behavior of MTs in solution.

The PB equation is derived by assuming that the concentration of an ionic species is given by a Boltzmann distribution

(1) $$\begin{array}{l} c=c_{s}\exp\frac{-ezV}{k_{B}T} \end{array}$$

where *c* is the local concentration of ions, *c*_*s*_ is the concentration of ions in the solution at equilibrium (when no external electric field is applied), *z* is the valence of the ion, *e* is the charge of an electron, *V* is the electrostatic potential, *k*_*B*_ is Boltzmann's constant, and *T* is the absolute temperature. The electrostatic potential is given by Poisson's equation

(2) $$\begin{array}{l} \nabla \cdot \left(\epsilon_{r}\nabla V\right)=-\frac{\rho }{\epsilon_{0}} \end{array}$$

where ρ is the charge density, ϵ_*r*_ is the relative permittivity (which is typically considered to be constant), and ϵ_0_ is the permittivity of free space. Assuming that the ions are the only charge carriers in the system, the charge density can be related to the concentrations of ionic species by

(3) $$\begin{array}{l} \rho =N_{A}\underset{i}{\sum }z_{i}ec_{i} \end{array}$$

where *N*_*A*_ is Avogadro's constant, *z*_*i*_ is the valence of the i'th ion, and *c*_*i*_ is the concentration of the i'th ion. Therefore, by combining Equations (1) and (2), we obtain the PB equation for electrostatic potential in an ionic solution

(4) $$\begin{array}{l} \nabla \cdot \left(\epsilon_{r}\nabla V\right)=-\frac{N_{A}e}{\epsilon_{0}}\underset{i}{\sum }z_{i}c_{s,i}\exp\frac{-ez_{i}V}{k_{B}T} \end{array}$$

where all quantities are as previously defined.

Because the PB equation represents a mean-field theory, it does not take into account ion-ion interactions or ion-size. A standard correction to account for the size of ions near the surface of the polyelectrolyte—where the concentration is the highest—is to enforce a distance of closest approach. We follow the terminology used in other biophysics papers and refer to this ion-exclusion region surrounding the polyelectrolyte as the Stern Layer (Chu et al., 2007; Silalahi et al., 2010; Kirmizialtin et al., 2012; Wang et al., 2014). We would like to clarify that—in contrast to how the Stern Layer is often defined—this layer does not contain any charge and does not account for absorption of ion charge onto the surface (which would decrease the effective charge of the polyelectrolyte). As defined in this work, the Stern Layer only corrects for the size of ions next to the surface of the polyelectrolyte. A number of studies on DNA have utilized Size Modified Poisson-Boltzmann (SMPB) equations, which consider the size of every ion in the system; unfortunately, the ion size values which yield results that agree with experiment are nonphysical. For example, the ion-sizes used in Chu et al. (2007)'s SMPB analysis of DNA are fit as experimental parameters and do not correspond to known hydration radii. Other authors have calculated the correct ion-size to use in SMPB models of DNA using molecular dynamics (MD) simulations (Kirmizialtin et al., 2012). Furthermore, despite the increased complexity of SMPB theories, a comparison of the prediction of ion distributions near a lipid membrane found that the SMPB equation was, in general, no more accurate than the PB equation with a Stern Layer correction (Wang et al., 2014).

The predictions of PB theory around DNA molecules have been compared to experimental measurements and more sophisticated theoretical models. The PB equation, SMPB equation, and MD simulations all give similar predictions for the total number of bound-ions (a far-field observable); the differences arise in estimates of local ionic concentrations (Kirmizialtin et al., 2012). Ionic concentrations near the polyelectrolyte are difficult to investigate experimentally; however, the surface concentrations predicted by PB have been compared to Monte-Carlo techniques and were found to have a relative error of 15–25% for monovalent ions and 25–30% for divalent ions (Pack et al., 1999). The PB equation is known to underestimate the concentration of ions at the surface of DNA; however, this can be mostly remedied for monovalent ions by accounting for a variable permittivity near the protein surface (Lamm and Pack, 1997; Kirmizialtin et al., 2012).

An excellent model for the variation in relative permittivity around a biological molecule, combining numerous known effects, was outlined by Lamm and Pack (1997). There are three main causes of variation in relative permittivity near a protein surface: (a) the geometry of the surface; (b) the local electric field of the protein (The Booth Effect); and (c) the effect of hydrated ions, which are far more concentrated close to the surface (Lamm and Pack, 1997). That equation is actually just accounting for the Booth effect, the relative permittivity, ϵ_*BE*_, is given by

(5) $$\begin{array}{l} \epsilon_{BE}=1.8+\left(\epsilon_{l}-1.8\right)L\left(0.08E\right) \end{array}$$

where the temperature is taken to be 298 K, ϵ_*l*_ is the local permittivity due to the geometry, *L* is the Langevin function defined by *L*(*x*) = 3[coth(*x*) − 1/*x*]/*x*, and *E* is the electric field in mV/Angstrom. Changes in relative permittivity due to ionic concentration are given by

(6) $$\begin{array}{l} \epsilon =\epsilon_{w}\left(1-\rho +3\alpha \rho \right)/\left(1+\rho /2\right) \end{array}$$

where ϵ_*w*_ is the bulk permittivity (the relative permittivity of water), α = ϵ_*K*_/2ϵ_*w*_, and ρ is the volume fraction of potassium ions given by

(7) $$\begin{array}{l} \rho =c\left[K^{+}\right]/\left(c\left[K^{+}\right]+15.9\left[M\right]\right) \end{array}$$

where *c*[*K*^+^] is the concentration of potassium ions and 15.9 [*M*] is the maximum concentration of potassium ions (Pack et al., 1999). To account for all causes of relative permittivity variation near a protein we can replace ϵ_*w*_ in Equation (6) with ϵ_*BE*_ in Equation (5).

Extensive work has gone into predicting counterionic condensation close to the surface of DNA molecules using PB theory. In this paper, we use PB theory to analyze MTs, predicting the surrounding electrostatic potential and ionic concentrations. The work discussed above has shown that the predictions of PB theory are relatively accurate for DNA, and initial comparisons between the predictions of MD simulations and PB theory for microtubules have shown excellent agreement (Shen and Guo, 2018). Using the predicted counterion concentrations we calculate MT electrical properties such as ionic conductivity, distance of electrostatic influence, and effective charge. Since Manning's theory is inapplicable at physiologically relevant ionic concentrations, our work will allow—to our knowledge for the first time—a relatively accurate determination of MT electrical parameters over a wide range of ionic concentrations.

The analysis is presented in the following fashion. First, we will describe the MT model and outline the calculations necessary to solve the system. Second, we will analyze the impact of different modifications to the Poisson-Boltzmann equation and investigate the accuracy of the analytical solution produced by linearizing the equation. Third, we will extend our calculations to a wide range of ionic concentrations and analyze how the local concentrations and potentials change as well as what the conductivity and effective charge of an MT are in different ionic concentration solutions.

## 3. Solving the Poisson-Boltzmann Equation

We begin by modeling an MT as an infinitely long, hollow cylinder with two separate surface charge densities. The inner and outer radii of the cylinder are 8.4 and 12.5 nm, respectively, and the surface charge densities are calculated assuming a solution pH of 7 (this assumption is made throughout our analysis, and all charges quoted will be for tubulin in a buffer of pH 7). The N-terminus of a tubulin dimer carries a charge of −5 e, which corresponds to an inner MT surface charge density of −0.025 *C*/*m*^2^ (calculated for the 3RYF structure of tubulin in the Protein Data Bank; Nawrotek et al., 2011). The outer surface of the MT has a charge of −25 e per dimer which corresponds to a surface charge density of −0.083 *C*/*m*^2^. These charge values do not include the C-termini, so they represent the physical characteristics of a subtilisin-digested MT (SMT) (Sackett et al., 1985; Shen and Guo, 2018).

The C-termini (CT) are modeled separately from the rest of the MT. Each CT is approximated as a cylinder 4 nm long, 1 nm wide, and carrying an electrostatic charge of −11e. Thus, the surface charge density of a CT is calculated to be −0.140 *C*/*m*^2^. Throughout the remainder of this paper CT are modeled as infinite cylinders with the specified surface charge density and radius. This approach neglects the CT's finite length; however, it reduces computational complexity and, as we shall see, provides results which compare well with experimental values.

The splitting of the MT structure into two cylindrical systems (SMTs and CTs) permits the utilization of cylindrical symmetry when solving the PB equation. In this analysis we only consider monovalent, symmetric ionic solutions (this simplifies calculations, but the same basic technique will apply to other buffers with the caveat that divalent ions are modeled more poorly by PB), so the PB equation (Equation 4) simplifies to

(8) $$\begin{array}{l} \frac{1}{r}\frac{d}{dr}\left(r\epsilon_{r}\frac{dV}{dr}\right)=\frac{2eN_{A}c_{s}}{\epsilon_{0}}\sinh\left(\frac{eV}{k_{B}T}\right) \end{array}$$

where *r* is the radial distance, ϵ_*r*_ is the relative permittivity, *V* is the electrostatic potential, *N*_*A*_ is Avogadro's number, *c*_*s*_ is the ionic concentration of bulk solution, ϵ_0_ is the permittivity of free space, *e* is the electronic charge, *k*_*B*_ is Boltzmann's constant, and *T* is the absolute temperature. When the electrostatic potential is less than the thermal voltage (*V*_*t*_ = −*e*/*k*_*B*_*T* ≈ −25 *mV*) this equation can be linearized, resulting in the expression

(9) $$\begin{array}{l} \frac{1}{r}\frac{d}{dr}\left(r\epsilon_{r}\frac{dV}{dr}\right)=\frac{2eN_{A}c_{s}}{\epsilon_{0}}\frac{eV}{k_{B}T} \end{array}$$

where all values are as previously defined. For clarity we will now refer to Equation (8) as the non-linear PB (NLPB) equation and Equation (9) as the linear PB (LPB) equation.

While the relative permittivity in the NLPB and LPB equations is often assumed to have a constant value, this assumption is not entirely accurate (as discussed in the section 2). We can account for the variation in relative permittivity using the work of Lamm and Pack (1997). Lamm and Pack found that, for cylinders, the effect of surface geometry on the local relative permittivity was negligible compared to the Booth and concentration effects, so the relative permittivity is calculated using Equations (5) and (6) and ϵ_*l*_ is taken to be the permittivity of water (Lamm and Pack, 1997). Accounting for a variable relative permittivity is one correction to the PB equation we consider, the other is the application of a Stern Layer to the boundary conditions for the equation.

We solve the PB equation both outside the MT and inside the lumen using Neumann boundary conditions. At the center of the lumen, the electric field is zero due to axial symmetry; the electric field is also zero infinitely far away from the outer surface of any cylinder (a CT or an SMT). Therefore, asymptotically:

(10) $$\begin{array}{l} {\frac{dV}{dr}}_{r=0,\infty }=0 \end{array}$$

At the surface of a charged cylinder, the electric field can be determined using the surface charge density and electromagnetic interface conditions. Then the electric field at the outer edge of the Stern Layer can be found by applying Gauss's law to the exclusion region. Therefore, the boundary condition for the outside of a charged cylinder (either an SMT or a CT) is given by

(11) $$\begin{array}{l} {\frac{dV}{dr}}_{r=R_{0}+d}=-\frac{\sigma_{o}}{\epsilon_{r}\epsilon_{0}}\frac{R_{0}}{R_{0}+d} \end{array}$$

where *R*_*o*_ is the outer radius of the cylinder, σ_*o*_ is the charge density of the outer surface, and *d* is the Stern Layer thickness or the distance of closest approach of hydrated ions. In this work, this value is taken to be 0.33 nm, which is the radius of a hydrated potassium ion (Israelachvili, 2011), the most common cation within the cytosol (Andersen, 2013). Thus, our model is further constrained to only apply to buffers where the cation is *K*^+^, the most abundant cation in living cells, although this is trivially extended by changing the value of *d* in this equation and the maximum concentration in Equation (7). Using the approach outlined above for the outer boundary condition, the boundary condition for the inside of a charged cylinder is found to be

(12) $$\begin{array}{l} {\frac{dV}{dr}}_{r=R_{i}-d}=\frac{\sigma_{i}}{\epsilon_{r}\epsilon_{0}}\frac{R_{i}}{R_{i}-d} \end{array}$$

where *R*_*i*_ is the inner radius of the cylinder, σ_*o*_ is charge density on the inner surface, and *d* is as defined.

Inherent in these boundary conditions, Equations (11) and (12), is an assumption of lumen electroneutrality. When applying the interface condition to the surfaces of the MT, we assume that the electric field inside the protein is zero, which is equivalent to assuming that the net charge of the lumen (the −5 e per dimer plus the charge of the counterions) is zero. The electroneutrality of charged cylindrical nanopores (which are modeled identically to our model of the lumen) has been verified by previous work, but only if the decrease in electric field over the Stern Layer is accounted for Lo and Chan (1994). As we have accounted for this factor, we assume electroneutrality and the validity of Equations (11) and (12)—this assumption was verified at the end of all calculations.

In this work, calculating the solution of the cylindrical NLPB Equation (8) is done numerically in COMSOL Multiphysics 5.5 (see Supplementary Material for details); however, the LPB equation (Equation 9), with a constant relative permittivity, can be solved analytically in cylindrical geometries. When the permittivity is constant Equation (9) can be re-written as a Bessel equation which has the standard solution

(13) $$\begin{array}{l} V\left(\frac{r}{\lambda_{D}}\right)=C_{i}I_{0}\left(\frac{r}{\lambda_{D}}\right)+C_{k}K_{0}\left(\frac{r}{\lambda_{D}}\right) \end{array}$$

where *I*_0_ is the zeroth modified Bessel function of the first kind; *K*_0_ is the zeroth modified Bessel function of the second kind; λ_*D*_ is the Debye length, which is given by $\lambda_{D}=\sqrt{\left(\epsilon_{0}\epsilon_{r}k_{B}T\right)/\left(2N_{A}c_{s}e^{2}\right)}$; and *C*_*i*_ and *C*_*k*_ are arbitrary constants. We can solve for *C*_*i*_ and *C*_*k*_ by applying the boundary conditions 10, 12, and 11. Doing so gives the following solution for the potential inside a cylinder of radius *R* and surface charge density σ

(14) $$\begin{array}{l} V\left(r\right)=\frac{\sigma \lambda_{D}}{\epsilon_{0}\epsilon_{r}}\frac{R}{R-d}\frac{I_{0}\left(r/\lambda_{D}\right)}{I_{1}\left(\left(R-d\right)/\lambda_{D}\right)} \end{array}$$

where *I*_1_ is the first modified Bessel function of the first kind and *d* is the distance of closest approach of the ions to the surface. The solution for the potential outside a cylinder of radius *R* and surface charge density σ is

(15) $$\begin{array}{l} V\left(r\right)=\frac{\sigma \lambda_{D}}{\epsilon_{0}\epsilon_{r}}\frac{R}{R+d}\frac{K_{0}\left(r/\lambda_{D}\right)}{K_{1}\left(\left(R+d\right)/\lambda_{D}\right)} \end{array}$$

where *K*_1_ is the first modified Bessel function of the second kind and all other quantities are previously defined. Therefore, when Equation (8) can be linearized to Equation (9) there is an analytical solution given by Equations (14) and (15). However, if *V* ≥ *V*_*t*_ then Equation (8) needs to be solved numerically. Numerical solutions were produced in COMSOL Multiphysics 5.5, and the requisite simulations are discussed in the Supplementary Material.

## 4. Calculating Local Potentials and Concentrations

**Figure 2:**
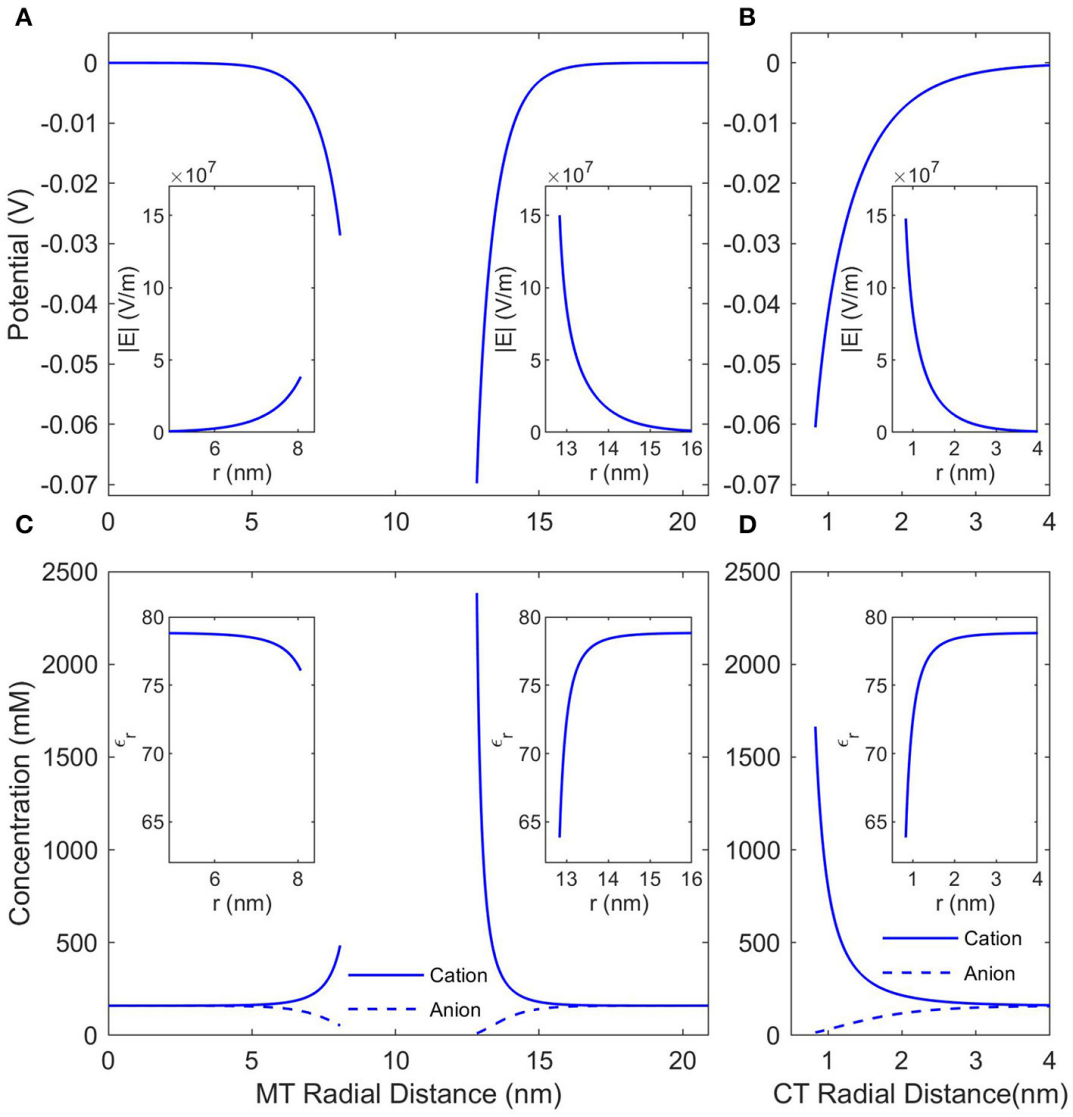
The counterionic concentrations and electrostatic potentials surrounding a microtubule when the buffer ionic concentration is 160 mM KCl. **(A)** Shows the potential profile near the inner and outer surfaces of an SMT; the radial distance is measured from the center of the microtubule, and the insets show the electric field strength as a function of the same radial distance. **(B)** Shows the potential profile near a single, isolated CT; the radial distance is measured from the center of the CT, and the inset shows the electric field strength as a function of the same radial distance. **(C,D)** Show the concentrations of anions and cations and the insets show the relative permittivity. The radial distances in **(C,D)** are the same as in **(A,B)**, respectively.

The local conditions around and inside an MT in a solution of KCl (or any other monovalent, symmetric buffer where *K*^+^ is the cation) with a concentration of 160 mM (the ionic strength of BRB80; Van den Heuvel et al., 2006) were calculated using the NLPB Equation (8) modified to account for the Stern Layer and the variable permittivity. Figure 2 presents the local electrostatic potentials, electric fields, anion and cation concentrations, and relative permittivities in the lumen, around the outer surface of an SMT, and around an isolated CT. As expected the local conditions differ dramatically from those of the buffer, and due the comparatively low charge density of the inner MT surface, the counterion condensation on the lumen is less extensive than that on the outer surface or the C-termini.

**Figure 3:**
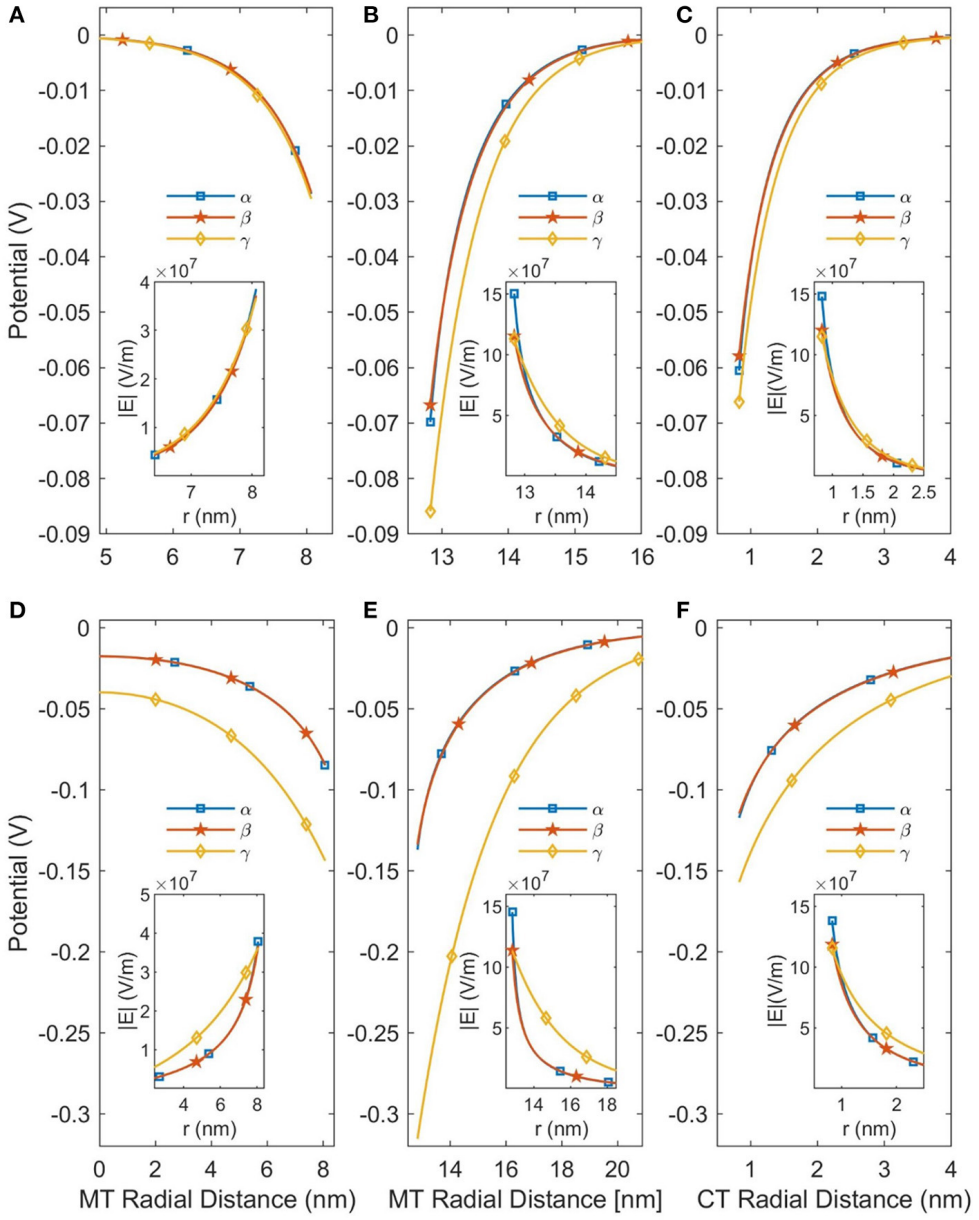
This figure demonstrates the effects of linearizing the PB equation and accounting for a variable permittivity. The curves labeled α are the predictions of the NLPB equation with a variable relative permittivity; the β curves are the NLPB equation with a constant relative permittivity; and the γ curves are the LPB equation with a constant relative permittivity. **(A–C)** Are for SMTs in a background ionic concentration of 160 mM KCl, while **(D–F)** are for a background ionic concentration of 10 mM. The SMT radial distance is the distance from the center of an SMT and the CT radial distance is the distance from the center of a CT.

In this work we considered two modifications to the PB equation, the Stern Layer and the Lamm and Pack model of variable permittivity. The Stern Layer modification is incorporated into all analyses presented in the paper. Not only is this modification computationally simple, but not including it results in significant differences to the calculated CT counterion concentration profiles as shown in the Supplementary Material. In contrast, the calculation of a variable permittivity is not computationally simple, and assuming a constant permittivity would simplify the analysis considerably. Therefore, we have analyzed the affect that accounting for a variable permittivity has on the solution, and these results are presented in Figure 3. Although the changes in permittivity shown in Figure 2 initially suggest that incorporating the variable permittivity is necessary for accurate results, assuming the permittivity to be constant does not result in a significant change in the predicted potential as seen in Figure 3. To increase the accuracy of our analysis we will consider the permittivity to be variable for the rest of this paper but assuming the permittivity to be constant is a completely acceptable approximation for future analyses.

However, using the NLPB equation is necessary to produce accurate predictions of the potential. As can be seen in Figure 3 the linear solutions are only approximately accurate when the buffer ionic concentration is 160 mM as the potential is already greater than *V*_*t*_. When the buffer ionic concentration decreases to 10 mM, the potential increases because the MT is less screened by ions and the linear solutions become completely inaccurate. Therefore, it is not possible to apply the approximations which lead to analytical solutions, and this system needs to be solved numerically—particularly at low (< 10 mM) buffer ionic concentrations.

## 5. Effects of Varying Buffer Concentration

**Figure 4:**
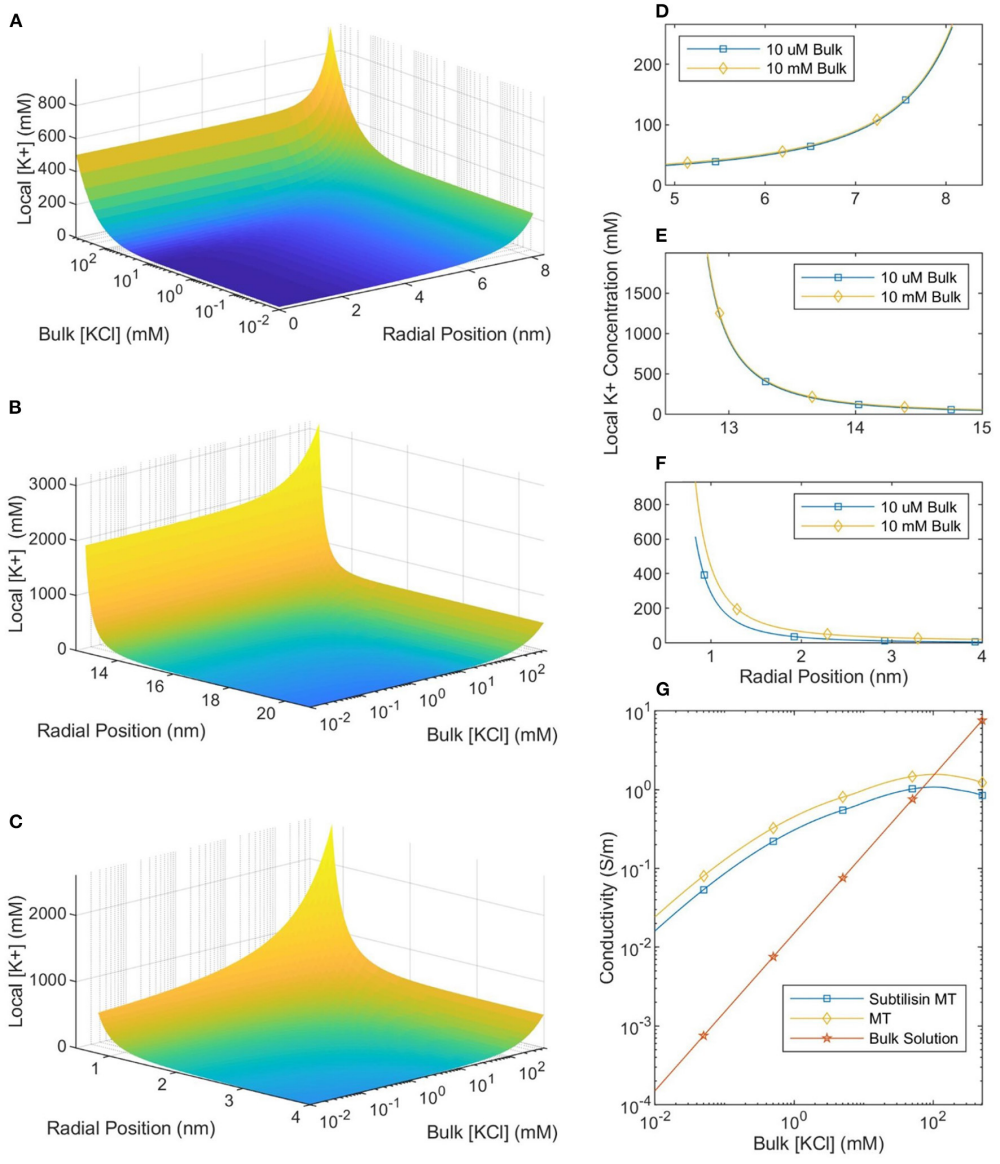
The local counterionic concentration—as a function of buffer ionic concentration—in the lumen **(A)**, around the outer SMT surface **(B)**, and around a CT **(C)**. The radial position in **(A,B)** is measured from the center of the SMT while the radial position in **(C)** is measured from the center of the CT. **(D–F)** Show the similarity of the counterionic concentration profiles—near the lumen, outer SMT surface, and CT surface, respectively—for MTs in 10 μM and 10 mM ionic buffer solutions. **(G)** Shows the conductivity of the bulk solution, an SMT-ion complex, and an MT-ion complex as a function of bulk solution ionic concentration.

The local ionic concentration profiles around an MT in a physiological ionic concentration buffer (100–500 mM) are dramatically different (Figure 4) to those around MTs in lower ionic concentration buffers (10 μM to 10 mM; typically used in experiments investigating MT electrical properties and MT nanodevice applications). The calculated ionic concentrations (the system was solved using the NLPB equation with the Stern Layer and variable permittivity both accounted for) near the surface of the protein are approximately constant for buffer ionic concentrations ranging from 10 μM to 10 mM. However, as the buffer ionic concentration increases further it reaches the same order of magnitude as the counterion concentration and large changes in counterion concentration are observed. Therefore, we can make a clear distinction between two types of buffers: low ionic concentration buffers and physiologically relevant (high ionic concentration) buffers.

Since ionic conductivity is proportional to the number of charge carriers, the local conductivity can be equated with the local ionic concentration. Because the local ionic concentration remains relatively constant for low ionic concentration buffers, the ratio between local and buffer conductivity will increase as the concentration decreases below 10 mM (see Figure 4). When the buffer ionic concentration is 10 μM there is a five orders-of-magnitude difference between the ionic concentration at the outer surface of the MT and in the buffer. This means that the MT-ion complex can be clearly distinguished as a separate system from the buffer, a highly conductive “wire” in a non-conductive medium. However, as the buffer ionic concentration increases by almost four orders of magnitude from 10 μM to 500 mM, the surface concentration only increases by a factor of ≈2. Therefore, at physiological ionic concentrations, there is much less of a distinction between the conductivity of the counterionic layer and the buffer.

To calculate the mean conductivity of an MT, we need to determine which counterions are considered part of the MT-ion complex. We begin by assuming that ions, both outside the MT and inside the lumen, with an electrostatic potential which is less than the thermal potential are “bound” to the MT. This assumption has been used successfully in work on DNA (Pack et al., 1999). We also assume that the molar conductivity of an ion along a equipotential surface is equal to the bulk molar conductivity (7.352*S* · *m*^2^/*mol* for *K*^+^ ions and 7.634*S* · *m*^2^/*mol* for *Cl*^−^ ions). Therefore, the axial MT conductivity can be calculated by integrating the local ionic conductivity over these “bound” ions and normalizing by the area. For SMTs we calculate the conductance of the counterionic layers “bound” to both the inner and outer surfaces and normalize by the total cross-sectional area of the MT and the bound ionic layers. When CTs are included in the calculation, we make the approximation that they can be treated as 13 infinite cylinders (C-termini are ≈ 4 nm long and separated by ≈ 4 nm along a protofilament) in parallel with the SMT and calculate the total conductance accordingly; this conductance is normalized in the same way. The calculated values of mean MT conductivity are plotted in Figure 4.

**Figure 5:**
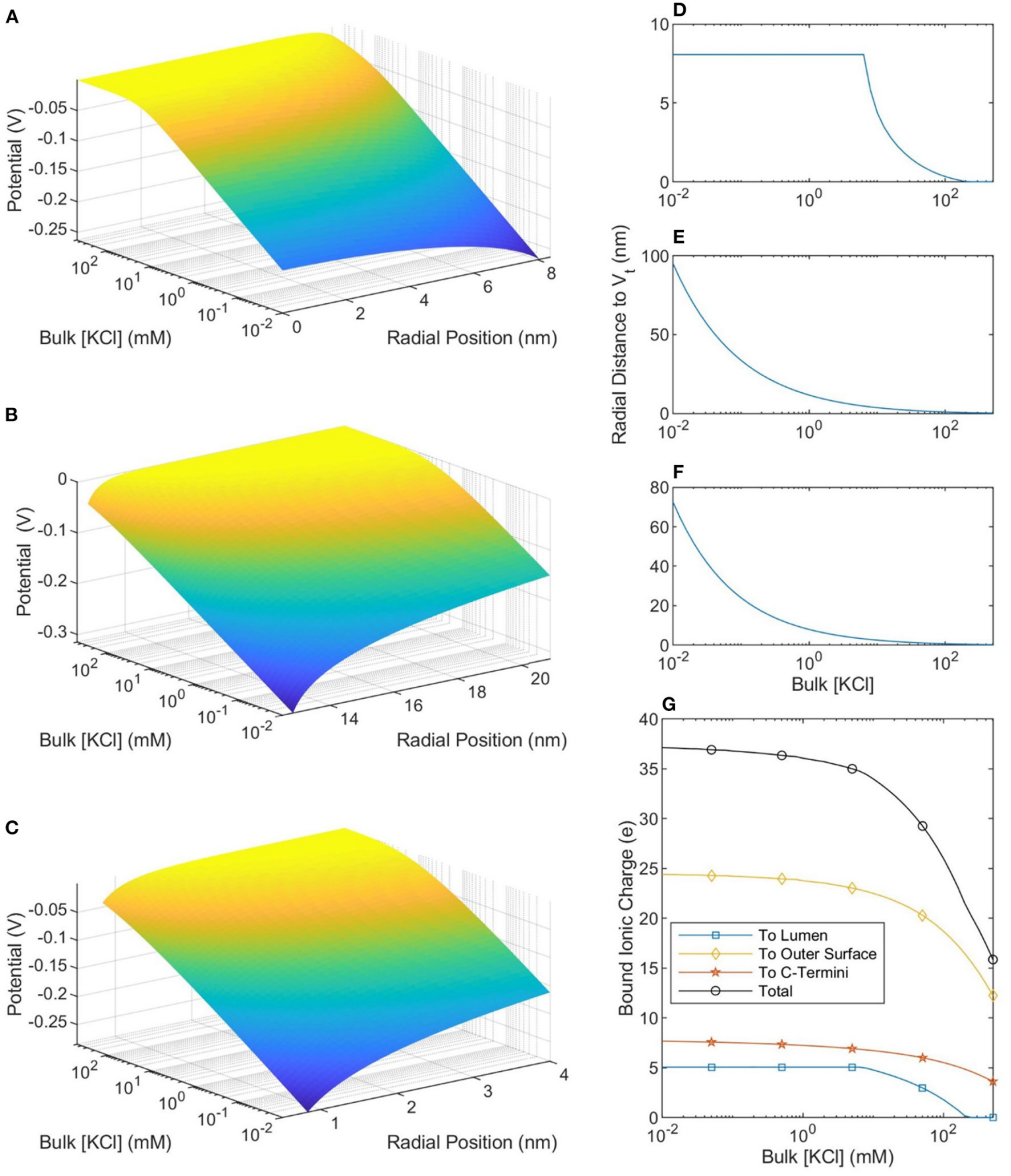
The potential profiles—as a function of buffer ionic concentration—in the lumen **(A)**, around the outer SMT surface **(B)**, and around a CT **(C)**. The radial position in **(A,B)** is measured from the center of the SMT while the radial position in **(C)** is measured from the center of the CT. **(D–F)** Show, respectively, the distance from the lumen, outer SMT surface, and CT surface to the thermal voltage. The maximum value in **(D)** is the radius of the lumen and at these points the entire lumen has a potential greater than the thermal potential. **(G)** Shows the total ionic charge within the thermal voltage potential, which can be considered the charge “bound” to the microtubule.

The size of, and number of charges in, the bound ionic layer (where |*V*| < *V*_*t*_) are shown in Figure 5. When the buffer ionic concentration is low, 10 μM, the counterionic layer extends 95 nm away from the outer SMT surface. In contrast, this value decreases to 0.22 nm at bulk ionic concentrations of 500 mM. When the buffer ionic concentration is large, the number of ions bound to the lumen is zero, as the magnitude of the potential at the Outer Helmholtz Plane is <25 mV. This is in stark contrast to the scenario at low buffer concentrations when the entire lumen is at a potential where the ions can be considered bound to the MT. These predictions raise interesting questions about the effective charge of an MT in solution. Figure 5 shows that at ionic concentrations below 10 mM, the net charge of the MT-ion complex has a stable value of ≈−15 e per heterodimer, whereas the net charge of the complex increases to −36 e per heterodimer at 501 mM. Therefore, the distinction we have already made between low and physiological ionic concentration buffers on the basis of ionic concentrations and conductivity is also meaningful when considering the net charge of the MT-ion complex.

## 6. Discussion

In this paper, we analyzed the basic electrical properties of MTs in different ionic concentration buffers. Previous studies—which calculated MT electrical parameters using Manning's theory of counterionic condensation—may not be accurate at physiological ionic concentrations, where the Debye length is low and the assumptions behind Manning's theory are more strained. Manning's theory is also limited by the fact that it only predicts properties which are observable in the far-field, such as net charge, and does not predict the structure of the condensed counterionic layer close to the MT surface. These limitations can be avoided by using the PB equation to predict counterion concentration. In this work, we numerically solve the NLPB equation, accounting for a variable permittivity and Stern's Layer, and are able to predict the counterion concentration profiles for MTs in buffers with a wide range of KCl concentrations, from 10 μM to 500 mM. These results allow us to investigate how MT electrical properties change with buffer ionic concentration. We then explicitly calculate the mean conductivity of, and total charge bound to, an MT as a function of ionic concentration. Our results demonstrate that the ionic concentration of the buffer is a critical parameter and that conclusions of theoretical and experimental papers which use a particular buffer ionic concentration should only be extended to other buffers with care.

As shown in Figure 4 there is a distinct difference between counterion profiles in low concentration (10 μM to 10 mM KCl) and physiological concentration (100–500 mM KCl) buffers. In low ionic concentration buffers, the counterion profiles are approximately invariant with respect to changes to the buffer concentration. In future work, this could be experimentally verified with the use of concentration sensitive fluorophores; however, this result is not unexpected. In low ionic concentration buffers, the Debye length is large enough for Manning's theory to be more applicable, and one of the predictions of Manning's theory is that counterion condensation is invariant to changes in the buffer ionic concentration. As we see in Figure 5, far-field parameters are also invariant at low bulk ionic concentrations, and the total number of ions bound to the MT is approximately constant when the buffer ionic concentration is lower than 10 mM. As buffer ionic concentration increases above 10 mM, there are drastic changes in local counterion concentration profiles and the total number of bound counterions. This observation has important implications for both medical research and nanodevice applications where ionic concentrations may vary either due to pathophysiology or in a controllable fashion, respectively.

The computed counterion condensation profiles were also used to calculate the mean conductivity of an MT as a function of bulk ionic concentration, and the results are presented in Figure 4. We can compare these predictions to measurements of single MT conductivity (Minoura and Muto, 2006). By approximating MTs as elliptical nanoparticles, Minoura and Muto (2006) used electroorientation measurements to determine that MT conductivity was 150 mS in a symmetric, monovalent solution with a KCl concentration of 10 μM and that CT cleavage (using Subtilisin) caused the conductivity to decrease by 36%. Notably, our calculated MT conductivity in a 10 μM solution is 14 mS, with a 66% decrease when the CT are not included in the model. These values are roughly consistent with the experimental measurements. Furthermore, we expect our calculated conductivity to be lower than that measured in an electroorientation experiment because we consider ions out to the thermal voltage radius to be part of the MT-ion complex. However, during electroorientation the MT-ion complex would be defined by the zeta potential (corresponding to the slip-plane). The zeta potential would likely have a greater magnitude than the thermal voltage and occur closer to the protein surface; thus, the mean conductivity of the MT-ion complex defined by the slip plane would be greater. Therefore, the model of mean MT conductivity presented here is consistent with measurements made in low ionic concentration buffers.

Our model also predicts an experimentally-observed change in MT conductivity as the buffer ionic concentration increases. As seen in Figure 4 there is a “crossover” ionic concentration, where MTs transition from being more conductive than the buffer, to being less conductive. This is due to the buffer conductivity increasing and the number of bound counter-ions decreasing while the total MT-ion complex cross-sectional area approaches a set value (the cross-sectional area of the MT). At the crossover ionic concentration, the size of the conductive counter-ionic cloud decreases such that the total MT conductivity is lower than that of bulk solution (this analysis assumes that any electronic conductivity of the protein is negligible compared to the ionic conductivity). Notably, experimental work appears to mirror this trend; measurements by Santelices et al. (2017) of MTs in a 20-fold diluted BRB80 buffer (with an ionic concentration of 8 mM) found the presence of MTs to increase conductivity. However, measurements by Kalra et al. (2020b) in a non-diluted BRB80 buffer (with an ionic concentration of 160 mM) demonstrated the opposite, with the presence of MTs decreasing sample conductivity. The results of this paper are consistent with these experiments, predicting that the addition of MTs should increase solution conductivity when the buffer ionic concentration is less than ≈ 100 mM (with greater differences being observed at lower ionic concentrations) and decrease solution conductivity when the buffer ionic concentration is greater. The corresponding crossover concentration for SMTs is predicted to be ≈ 70 mM. Interestingly, constructing nanodevices based on MTs immersed in a solution with a variable ionic concentration could lead to controllable ionic conduction flows.

Notably, there are two types of MT conductivity to distinguish here: one is the mean conductivity of the entire MT, which defines how MTs change the conductivity of a solution and has been discussed in the previous two paragraphs; the other is the local conductivity of the counterionic cloud, which would effect the propagation of theorized, and previously mentioned, ionic solitons along the MT (Priel and Tuszyński, 2008; Satarić et al., 2009; Sekulić et al., 2011; Sekulić and Satarić, 2012). As seen in Figure 4 and discussed in section 5, the counterionic concentration—and, therefore, the conductivity—profiles remain stable at low solution concentrations, but change at higher ones. Therefore, it is conceivable that signal propagation along MTs in cells could vary significantly with changes in intracellular ionic concentration, which could occur locally, e.g., near the cell nucleus or the mitochondria. However, in experiments at low ionic concentrations—and in nanodevices operating in low ionic concentration buffers—signal propagation would be relatively immune to changes in the buffer ionic concentration. There are too few experimental measurements of ionic signal propagation to make any comparisons, but we predict significant differences between signal propagation along MTs in low and physiological ionic concentration buffers.

Our results provide—to our knowledge for the first time—insight into the behavior of MT counterions at physiologically relevant ionic concentrations. When the ionic concentration of the buffer is low, there is a difference of several orders of magnitude between the bulk ionic concentration and the ionic concentration in the vicinity of the MT surface (see Figure 4). Thus, on the basis of conductivity, we can clearly distinguish between bulk solution and the MT-ion complex. However, at physiologically relevant ionic concentrations, the MT counterion concentration is only a few times that of bulk. Notably, we do not see any evidence of a “depleted layer” separating condensed counterions from the solution as was postulated in previous works where it was argued that a depleted region would contain ionic signals which propagated along the MT (Priel and Tuszyński, 2008). Note that we have not calculated the radial conductivity of the counterionic cloud (the conductivity “seen” by signals traveling radially to the MT) as it is unclear what the molar conductivity would be for ions which are moving across equipotential lines. This calculation, and an analysis of signal propagation along MTs at higher concentrations, should be a thrust of future work. Because our results do not support the assumption of a depleted layer separating counterions from the solution, ionic signal propagation along MTs may not be as stable as predicted by previous transmission line models (which assumed the presence of a depleted layer). Thus, a rigorous analysis of the radial conductivity is required to analyze how propagating ionic signals will interact with the surrounding buffer.

Further measurements of MT electrical properties should be a focus of future experimental work. Direct experimental measurements of the counterion condensation around MTs would also be useful to confirm or refute the predictions in this paper and, more generally, the accuracy of PB theory as applied to MTs. One known flaw in PB theory is that it does not account for ion-ion interactions. Modifications to the PB equation to account for ion-ion interactions have been proposed (Forsman, 2004) and should be incorporated into future work. We also suggest further theoretical work investigating how MT electrical properties will differ when an MT is interacting with an interface. Previously studied effects of buffer ionic concentration changes on polyelectrolyte adsorption to the interface would need to be accounted for de Carvalho et al. (2016) and Cherstvy and Winkler (2012), but a greater understanding of MT electrical properties on interfaces would pave the way for new experimental possibilities. A theoretical analysis of radial conductivity is also an important component of future work necessary to improve our model of MT electrical properties. Finally, signal propagation due to synchronous CT oscillation should be investigated. As seen in Figure 2, the potential well of a single CT overlaps with that of the nearest adjacent CT at buffer ionic concentrations of 160 mM, raising the possibility of coordinated CT motion, which may give rise to synchronized electro-mechanical waves propagating along the MT surface involving CT motion. Our work did not consider interactions between multiple CT or between CT and the MT body, nor did it extend current transmission line models of MTs to higher buffer ionic concentrations. In the future, these extensions would be important for the development of a dynamic model of the electrical properties of a single MT.

## 7. Conclusion

The results in this work illustrate the influence of the buffer on the electrical properties of MTs. Counterionic condensation around MTs is strongly dependent on the buffer ionic concentration, which can be divided into two distinct regimes: the low concentration regime (10 μ*M* to 10 *mM*), and the physiological concentration regime (100–500mM). MTs in these regimes have marked differences in local and far-field counterionic condensation parameters which have been explored here in depth and should be considered in future biological and nano-device research. We used the counterion condensation to calculate the conductivity of an MT as a function of buffer concentration and compared our results with previous experimental work. Importantly, our work predicts that MTs increase solution conductivity when the buffer ionic concentration is <100 mM but decrease it otherwise. These results demonstrate that the buffer ionic concentration is a critical parameter in determining MT electrical characteristics. Thus, our work provides insight into the bioelectrical properties of MTs and the biophysical properties of the cell.

## Data Availability Statement

Publicly available datasets were analyzed in this study. This data can be found at: All the data are contained in the article.

## Author Contributions

BE and SP performed the simulations. BE, SP, AK, VR, KS, and JT analyzed the data, edited, revised, and finalized the manuscript. BE wrote the original draft. JT and KS provided funding and computational resources. JT supervised the project. All authors contributed to the article and approved the submitted version.

## Conflict of Interest

The authors declare that this study received funding from Novocure Inc. The funder was not involved in the study design, collection, analysis, interpretation of data, the writing of this article or the decision to submit it for publication.

## Abbreviations

CT: C-termini
MT: microtubule
SMT: subtilisin-cleaved microtubule
PB: Poisson-Boltzmann
NLPB: non-linear Poisson-Boltzmann
LPB: linear Poisson-Boltzmann
MD: molecular dynamics.

## References

[B1] Andersen O. (2013). Cellular electrolyte metabolism. Encyclop. Metalloprot. 580–587. 10.1007/978-1-4614-1533-6_223

[B2] Cantero M. d. R. Perez P. L. Scarinci N. Cantiello H. F. (2019). Two-dimensional brain microtubule structures behave as memristive devices. Sci. Rep. 9:12398. 10.1038/s41598-019-48677-1 31455820 PMC6711987

[B3] Cantero M. d. R. Villa Etchegoyen C. Perez P. L. Scarinci N. Cantiello H. F. (2018). Bundles of brain microtubules generate electrical oscillations. Sci. Rep. 8:11899. 10.1038/s41598-018-30453-2 30093720 PMC6085364

[B4] Cherstvy A. G. Winkler R. (2012). Polyelectrolyte adsorption onto oppositely charged interfaces: image-charge repulsion and surface curvature. J. Phys. Chem. B 116, 9838–9845. 10.1021/jp304980e 22794191

[B5] Chu V. B. Bai Y. Lipfert J. Herschlag D. Doniach S. (2007). Evaluation of ion binding to DNA duplexes using a size-modified Poisson-Boltzmann theory. Biophys. J. 93, 3202–3209. 10.1529/biophysj.106.099168 17604318 PMC2025650

[B6] de Carvalho S. J. Metzler R. Cherstvy A. G. (2016). Critical adsorption of polyelectrolytes onto planar and convex highly charged surfaces: the nonlinear Poisson-Boltzmann approach. New J. Phys. 18:083037. 10.1088/1367-2630/18/8/083037

[B7] Forsman J. (2004). A simple correlation-corrected Poisson-Boltzmann theory. J. Phys. Chem. B 108, 9236–9245. 10.1021/jp049571u

[B8] Freedman H. Rezania V. Priel A. Carpenter E. Noskov S. Y. Tuszynski J. A. (2010). Model of ionic currents through microtubule nanopores and the lumen. Phys. Rev. E 81:051912. 10.1103/PhysRevE.81.051912 20866266

[B9] Gittes F. Mickey B. Nettleton J. Howard J. (1993). Flexural rigidity of microtubules and actin filaments measured from thermal fluctuations in shape. J. Cell Biol. 120, 923–934. 10.1083/jcb.120.4.923 8432732 PMC2200075

[B10] Gruziel M. Grochowski P. Trylska J. (2008). The Poisson-Boltzmann model for tRNA: assessment of the calculation set-up and ionic concentration cutoff. J. Comput. Chem. 29, 1970–1981. 10.1002/jcc.20953 18432617 PMC2599918

[B11] Isozaki N. Ando S. Nakahara T. Shintaku H. Kotera H. Meyhöfer E.. (2015). Control of microtubule trajectory within an electric field by altering surface charge density. Sci. Rep. 5:7669. 10.1038/srep07669 25567007 PMC4286733

[B12] Israelachvili J. N. (2011). Chapter 4: - Interactions involving polar molecules, in Intermolecular and Surface Forces, 3rd Edn, ed Israelachvili J. N. (San Diego, CA: Academic Press), 71–90. 10.1016/B978-0-12-375182-9.10004-1

[B13] Kalra A. P. Eakins B. B. Patel S. D. Ciniero G. Rezania V. Shankar K.. (2020a). All wired up: an exploration of the electrical properties of microtubules and tubulin. ACS Nano 14, 16301–16320. 10.1021/acsnano.0c06945 33213135

[B14] Kalra A. P. Patel S. D. Bhuiyan A. F. Preto J. Scheuer K. G. Mohammed U.. (2020b). Investigation of the electrical properties of microtubule ensembles under cell-like conditions. Nanomaterials 10:265. 10.3390/nano10020265 32033331 PMC7075204

[B15] Kalra A. P. Patel S. D. Eakins B. B. Riddell S. Kumar P. Winter P.. (2021). Revealing and attenuating the electrostatic properties of tubulin and its polymers. Small 17:2003560. 10.1002/smll.202003560 33295102

[B16] Kirmizialtin S. Silalahi A. R. J. Elber R. Fenley M. O. (2012). The ionic atmosphere around A-RNA: Poisson-Boltzmann and molecular dynamics simulations. Biophys. J. 102, 829–838. 10.1016/j.bpj.2011.12.055 22385854 PMC3283773

[B17] Lamm G. Pack G. R. (1997). Calculation of dielectric constants near polyelectrolytes in solution. J. Phys. Chem. B 101, 959–965. 10.1021/jp9623453

[B18] Lamm G. Pack G. R. (2010). Counterion condensation and shape within Poisson-Boltzmann theory. Biopolymers 93, 619–639. 10.1002/bip.21421 20213767

[B19] Lo W. Y. Chan K.-Y. (1994). Poisson-Boltzmann calculations of ions in charged capillaries. J. Chem. Phys. 101, 1431–1434. 10.1063/1.467767

[B20] Manning G. S. (1969). Limiting laws and counterion condensation in polyelectrolyte solutions I. Colligative properties. J. Chem. Phys. 51, 924–933. 10.1063/1.1672157

[B21] Minoura I. Muto E. (2006). Dielectric measurement of individual microtubules using the electroorientation method. Biophys. J. 90, 3739–3748. 10.1529/biophysj.105.071324 16500962 PMC1440755

[B22] Nawrotek A. Knossow M. Gigant B. (2011). The determinants that govern microtubule assembly from the atomic structure of GTP-tubulin. J. Mol. Biol. 412, 35–42. 10.1016/j.jmb.2011.07.029 21787788

[B23] O'Shaughnessy B. Yang Q. (2005). Manning-Oosawa counterion condensation. Phys. Rev. Lett. 94:048302. 10.1103/PhysRevLett.94.048302 15783607

[B24] Pack G. R. Wong L. Lamm G. (1999). Divalent cations and the electrostatic potential around DNA: Monte Carlo and Poisson-Boltzmann calculations. Biopolymers 49, 575–590. 10.1002/(SICI)1097-0282(199906)49:7<575::AID-BIP4>3.0.CO;2-J 10226502

[B25] Priel A. Ramos A. J. Tuszynski J. A. Cantiello H. F. (2006). A biopolymer transistor: electrical amplification by microtubules. Biophys. J. 90, 4639–4643. 10.1529/biophysj.105.078915 16565058 PMC1471843

[B26] Priel A. Tuszyński J. A. (2008). A nonlinear cable-like model of amplified ionic wave propagation along microtubules. Europhys. Lett. 83:68004. 10.1209/0295-5075/83/68004

[B27] Ramanathan G. V. (1983). Statistical mechanics of electrolytes and polyelectrolytes. III. The cylindrical Poisson-Boltzmann equation. J. Chem. Phys. 78, 3223–3232. 10.1063/1.445239

[B28] Sackett D. L. Bhattacharyya B. Wolff J. (1985). Tubulin subunit carboxyl termini determine polymerization efficiency. J. Biol. Chem. 260, 43–45. 10.1016/S0021-9258(18)89688-0 3965457

[B29] Santelices I. B. Friesen D. E. Bell C. Hough C. M. Xiao J. Kalra A.. (2017). Response to alternating electric fields of tubulin dimers and microtubule ensembles in electrolytic solutions. Sci. Rep. 7:9594. 10.1038/s41598-017-09323-w 28851923 PMC5574899

[B30] Satarić M. Ilić D. Ralević N. Tuszynski J. A. (2009). A nonlinear model of ionic wave propagation along microtubules. Eur. Biophys. J. 38, 637–647. 10.1007/s00249-009-0421-5 19259657

[B31] Sekulić D. L. Satarić B. M. Tuszynski J. A. Satarić M. V. (2011). Nonlinear ionic pulses along microtubules. Eur. Phys. J. E 34:49. 10.1140/epje/i2011-11049-0 21604102

[B32] Sekulić D. L. Satarić M. V. (2012). Microtubule as nanobioelectronic nonlinear circuit. Serbian J. Electr. Eng. 9, 107–119. 10.2298/SJEE1201107S

[B33] Shen C. Guo W. (2018). Ion permeability of a microtubule in neuron environment. J. Phys. Chem. Lett. 9, 2009–2014. 10.1021/acs.jpclett.8b00324 29617570

[B34] Silalahi A. R. Boschitsch A. H. Harris R. C. Fenley M. O. (2010). Comparing the predictions of the nonlinear Poisson- Boltzmann equation and the ion size-modified Poisson- Boltzmann equation for a low-dielectric charged spherical cavity in an aqueous salt solution. J. Chem. Theory Comput. 6, 3631–3639. 10.1021/ct1002785 22723750 PMC3378335

[B35] Stigter D. (1995). Evaluation of the counterion condensation theory of polyelectrolytes. Biophys. J. 69, 380–388. 10.1016/S0006-3495(95)79910-6 8527651 PMC1236262

[B36] Tuszynski J. A. Friesen D. Freedman H. Sbitnev V. I. Kim H. Santelices I.. (2020). Microtubules as sub-cellular memristors. Sci. Rep. 10:2108. 10.1038/s41598-020-58820-y 32034179 PMC7005844

[B37] Van den Heuvel M. De Graaff M. Lemay S. Dekker C. (2007). Electrophoresis of individual microtubules in microchannels. Proc. Natl. Acad. Sci. U.S.A. 104, 7770–7775. 10.1073/pnas.0608316104 17470799 PMC1876522

[B38] Van den Heuvel M. G. De Graaff M. P. Dekker C. (2006). Molecular sorting by electrical steering of microtubules in Kinesin-coated channels. Science 312, 910–914. 10.1126/science.1124258 16690866

[B39] van Eunen K. Bakker B. M. (2014). The importance and challenges of *in vivo*-like enzyme kinetics. Perspect. Sci. 1, 126–130. 10.1016/j.pisc.2014.02.011

[B40] Van Eunen K. Bouwman J. Daran-Lapujade P. Postmus J. Canelas A. B. Mensonides F. I.. (2010). Measuring enzyme activities under standardized *in vivo*-like conditions for systems biology. FEBS J. 277, 749–760. 10.1111/j.1742-4658.2009.07524.x 20067525

[B41] Wang N. Zhou S. Kekenes-Huskey P. M. Li B. McCammon J. A. (2014). Poisson-Boltzmann versus size-modified Poisson-Boltzmann electrostatics applied to lipid bilayers. J. Phys. Chem. B 118, 14827–14832. 10.1021/jp511702w 25426875 PMC4280115

