# Supplementary material for: Modeling Microtubule Counterion Distributions and Conductivity Using the Poisson-Boltzmann Equation
Source: Front Mol Biosci. 2021 Mar 25;8:650757. doi: 10.3389/fmolb.2021.650757 (PMC8027483; doi:10.3389/fmolb.2021.650757)
Supplement: Supplementary file 1 [file Presentation_1.pdf]

Supplementary Material

1 Supplementary Information for Microtubule Model

Figure 1 in the main text shows a structural illustration of an MT and its constituent heterodimers. A 3-dimensional rendering of an MT is shown in (A). This model is constructed using tubulin heterodimer building blocks as shown in (B). The template structure of the tubulin used is 1JFF (PMID:11700061), this particular model shows a bovine stabilized tubulin heterodimer with a taxol ligand and is shown in schematic 1 (B). The 1JFF tubulin conformation is used as it closely resembles the tubulin dimers bound in an MT-like arrangement instead of stand-alone heterodimers.

Note, we can alter the conformation of the C-termini in the alpha-tubulin and beta-tubulin monomers *via* the MOE software (Molecular Operating Environment; Chemical Computing Group, Montréal, Quebéc, Canada). For purposes of illustration, we do not consider this and simply visualize the readily available 1JFF model. This is done by opening the structure in the VMD software (Visual Molecular Dynamics, University of Illinois at Urbana Champaign) and using the in-built rendering function *via* a quicksurf method consisting of a 1-unit grain size. This rendering is then saved and imported into the SpaceClaim software (SpaceClaim Corporation, Concord, Massachusetts) as a faceted body. Here, a 0.1 nm shrinkwrap is applied to the imported 1JFF model to yield an atomically accurate solid body model of the tubulin heterodimer.

We can utilize this solid body tubulin model to construct the MT-like structure as shown in schematic 1 (A). The model is then imported into the Fusion 360 software (Autodesk Fusion 360, San Rafael, California), where a 13-unit ring of tubulin heterodimers is constructed. This ring structure has an inner diameter of 14 nm, and the tubulin heterodimers are oriented such that all the alpha-tubulins in the ring are adjacent and also all the beta-tubulins are adjacent. To visualize this more clearly, consider looking head-on onto the ring. Here, we see a ring of 13 alpha-tubulins, with a ring of 13 beta-tubulins directly behind the first ring. In this setup, all existing C-termini protrude outwards from the main ring. After this ring-like structure is created, we incorporate the helical nature of the MT by using the coil tool to introduce a helical path dependence. The helical path is set to yield a separation distance of 8 nm between each full rotation. Furthermore, this helical pattern is then repeated and rendered in Fusion 360 to yield the depiction of an MT as shown in schematic 1 (A).

It is important to note that this shrink-wrapped schematic of an MT is not conformationally accurate; however, proper dimensions of a tubulin heterodimer have been considered in the process of obtaining the solid-body model. Conformational inaccuracy stems from joining solid-body heterodimers instead of converting a full protein representation of an MT into a solid body. Hence, despite this shortcoming, the model is a reasonable approximation for a typical MT.

Moreover, if the work presented in this article is to be extended into a 3-dimensional simulation, then a solid-body model similar to that presented here is required to carry out the simulations in the COMSOL Multiphysics software (COMSOL Inc, Burlington, Massachusetts). In addition, the MOE software can be used to obtain a tubulin heterodimer structure with a varying C-terminus conformation: allowing for a more comprehensive study to be conducted that would showcase the range and versatility of the C-termini.

2 Supplementary Information for Numerical Simulations

The numerical simulations were performed using the Poisson Boltzmann Equation (PBE) on a 1D axisymmetric domain. The derivation of the PBE, the relevant boundary conditions, and the formulas describing the variable permittivity are presented in the main text. The resulting equations are solved numerically with the use of COMSOL 5.5. Here, the chronology of the simulations/results in the main text will not be strictly adhered to for the sake of clarity.

On a 1D axisymmetric domain, the PBE can be simplified, as is done in the main text, to yield Eq. 8. This is the nonlinear PBE (NLPBE), which can be solved with the boundary conditions given by Eq. 11 and Eq. 12. For each simulation performed, we divide the MT into 3 regions: the MT inner surface, the MT outer surface, and the c-termini. If we consider looking at the MT from its angular axis, we can visualize the regions of interest and assign them corresponding values corresponding to their radial position. The inner MT surface lies at 8.4 nm, and the outer surface lies at 12.5 nm. We assign the inner and outer surface charge density values of -0.025 C/m^2 and -0.083 C/m^2, respectively. These values do not account for the C-termini as this region will be analyzed separately.

Now, we can start building the model in COMSOL. The inner and outer radii and surface charge densities are specified as simulation parameters. A temperature of 300 K and a Stern layer thickness of d = 0.33 nm, corresponding to the radius of a hydrated potassium ion, are also specified. The procedure for solving the system inside the lumen is outlined below.

To solve the system inside the lumen, the radial domain is defined from 0 nm to R_i – d = 8.07 nm. For simulations where the relative permittivity is considered to be constant, we calculate the value of the relative permittivity using Eq.'s 6 and 7 from the main text and let the K^+ concentration in Eq. 7 be that of the buffer solution. Under the component definitions, we define three variables over the entire domain to organize the simulations. The first is alpha, as defined in Eq. 6, and the second and third are Eq. 6 and Eq. 7, respectively.

For simulations where the relative permittivity is considered to be variable, we calculate the value of the relative permittivity using Eq.'s 5-7 in the main text. Here, the K^+ concentration in Eq. 7 is changed from the buffer ionic concentration to the local concentration in the ionic double layer given by $cC = Cs \cdot e^{-\frac{eV}{k_B T}}$, where Cs is taken to be the buffer K^+ concentration and cC is the local K^+ concentration. We also add a variable defining ϵ_{BE}, given by Eq. 5, to the simulation, and this requires defining additional variables corresponding to the Langevin function and the E-field (given by $|\nabla V|$). These are all the variables and functions needed to explicitly define Eq. 5, Eq. 6, and Eq.7 from the main text. Note that Eq. 6 is what gives the final value of the varying permittivity (ϵ_v).

Note that the relative permittivity depends on the **E** field as defined by Eq. 5 in the main text, and for distances far from the surface where **E** ~ 0 we observe significant numerical error in the calculation of ϵ_r if the effect of the **E** field is included in the calculation, *i.e.*, if ϵ_w in Eq. 6 is taken to be $\epsilon_w = \epsilon_{BE}$. Therefore, we split the computational domain into two intervals: a 'near' permittivity interval ranging from 0.1 nm to R_i-d nm where the **E** field has a significant effect on the permittivity and ϵ_w in Eq. 6 is replaced with ϵ_{BE} from Eq. 5, and a 'far' permittivity interval ranging from 0 nm to 0.1 nm where the **E** field does not have a significant effect on the permittivity and ϵ_w in Eq. 6 can be

taken to be the relative permittivity of water. The positions of the near and far permittivity regions are chosen to ensure continuity of the relative permittivity

Again, note that these dimensions are along the radial axis of the MT. Next, a coefficient form PDE is selected as the physics solver over the entire 1D domain and the study is assumed to be stationary. This amounts to a diffusion coefficient of -1, a convection coefficient of

$$\frac{1}{x}+\frac{1}{\epsilon_r}\frac{d\epsilon_r}{dx}$$

(which simplifies to 1/x [1/m] when the relative permittivity is constant), and a source term equating to the right-hand side of Eq. 8 divided by the relative permittivity (whether it is variable or constant). All other coefficients are taken to be 0. Now the boundary conditions are applied. A zero-flux condition is applied at the center of the MT and a flux source is applied at the inner MT surface boundary. This is the right-hand side of Eq. 12. In addition, initial values for the potential and its time derivative are applied over the entire domain. These values are $-50 \cdot I_0(1, \frac{x}{R_i})$ [mV] and 0 [V/s], respectively; they are initial guesses stemming from the analytic solution and lead to a convergent simulation. Furthermore, a custom mesh is applied on the domain with a maximum element size of 0.005 nm and a maximum element growth rate of 1.1. Finally, a stationary study is conducted, and yields results of potential versus radial distance that are then exported and post-processed in Matlab (MATLAB, Natick, Massachusetts).

To solve the system outside MT surface, the radial domain is defined over R_0 + d = 12.83 nm to 100 nm, and the boundary conditions discussed in the main text are applied. The simulation is set up in much the same way except now when the permittivity is considered to be variable, the near-field permittivity interval is set to range from R_0+d nm to 20 nm, and the 'far-field' permittivity interval ranges from 20 nm to 100 nm. The zero-flux condition is applied to the boundary corresponding to the bulk solution and the outer surface condition is set to that described in Eq. 11. Lastly, the initial value across the domain is set to $-50e^{-\frac{x-R_0}{10\,[nm]}}$ [mV].

The final region to consider here is the C-termini region. This simulation is the same as that previously described except with a different protein outer radius (R_{ct} = 0.5 nm) and charge density. The radial domain is taken to range from R_{ct} + d = 0.83 nm to 100 nm, and the surface charge on the termini is taken to be -0.14 C/m^2, as justified in the main text. The geometry differs here by segmentation as well. The near-field permittivity interval is taken to be from R_{ct} +d nm to 10 nm, and the far-field permittivity interval is taken to range from 10 nm to 50 nm. Moreover, the bulk solution boundary is set to have zero flux and the entire domain is set to have initial value of $-50e^{-\frac{x-R_{ct}}{10\,[nm]}}$ [mV]. For the c-termini, the surface charge condition is identical to that of Eq. 11, except with σ_0 and R_0 replaced with σ_{ct} and R_{ct}, respectively.

We have now setup and solved the NLPB equation for when the permittivity remains constant and when it varies. The simulation setups described up till here yield the data shown in Figures 1 and 2. Now to vary the bulk ion concentration, we simply add a parameter sweep for the 'Cs' variable (which represents the buffer ionic concentration as mentioned above) in the study of the model. For the data shown in Figures 3 and 4, this ion concentration variable is swept from $10^{\log_{10} 0.01}$ to $10^{\log_{10} 505}$ in intervals of $10^{0.1}$ [mol/m^3].

There is a slight additional complexity when the bulk ionic concentration is considered to vary. Depending upon the range of the ‘Cs’ sweep the boundary between the far-field and near-field permittivity regions may need to be adjusted. This is because for different bulk ion concentrations there are different radial positions at which the **E** field (a) no longer strongly effects the relative permittivity and (b) begins to cause numerical errors in the value of the relative permittivity. Therefore, the boundary between the ‘far-field’ and ‘near-field’ relative permittivity regions may need to be adjusted or even defined as a function of the ionic concentration.

Simulation setups for numerically solving the NLPB equation have been presented. It is important to compare these results with other standard techniques used to solve the NLPB equation. The method of interest is solving via an analytic solution. This is done by first linearizing Eq. 8 to Eq. 9. To solve analytically, we need to assume the permittivity is constant. Implementing this, we see that Eq. 9 is a Bessel equation which has the standard solution as shown by Eq. 13. Using the boundary conditions, we can obtain general closed-form expressions for the potential in the region inside and outside of a cylinder with a given radius and charge density. Plotting Eq. 14 and Eq. 15, allows comparison with the NLPB equation solved with correction factors accounting for ion hydration and other similar effects which have been neglected in prior works. The differences between these approaches are shown in Figure 2. The simulation setups described here are used to obtain the potential and concentration data in the three regions of interest around a MT. Only the simulation for the lumen region is described using all methods – a similar process extends to the other regions of interest.

Note that Matlab scripts are used to post-process the electric potential data obtained from COMSOL. This data is used to determine the electric field intensity, ion concentration, and consistency checks in the theory by checking for electroneutrality in the lumen region. Intermediate consistency checks consist of verification of the variation of the permittivity as a function of distance.

3 Supplementary Information for the Stern Layer Modification

The Stern Layer is incorporated into every simulation for which results are presented in the main text. As discussed in the main text, this is because (a) modifying the boundary conditions to account for the Stern Layer is computationally simple and (b) without the Stern Layer the predicted concentration at the surface of the CT becomes unphysically large. This is easily demonstrated by modifying our simulations to remove the Stern Layer.

The Stern Layer can be removed by setting $d = 0$ in all simulations. Because $R \gg d$ for the SMT, this modification makes only a small difference in the predicted counterion concentrations near the inner and outer SMT surfaces (approximately 5%). However, for the CT, $R \approx d$; therefore, the removal of the Stern Layer makes a significant difference in the predicted counterion concentrations. These counterion concentration plots are shown in Fig. S1 and Fig. S2. Due to increased screening caused by the large counterion concentration at the CT surface, the predicted counterion concentration at 0.83 nm, where the Stern Layer would be, is half that of the simulations where the Stern Layer is included.

3.1 Supplementary Figures for the Stern Layer Modification

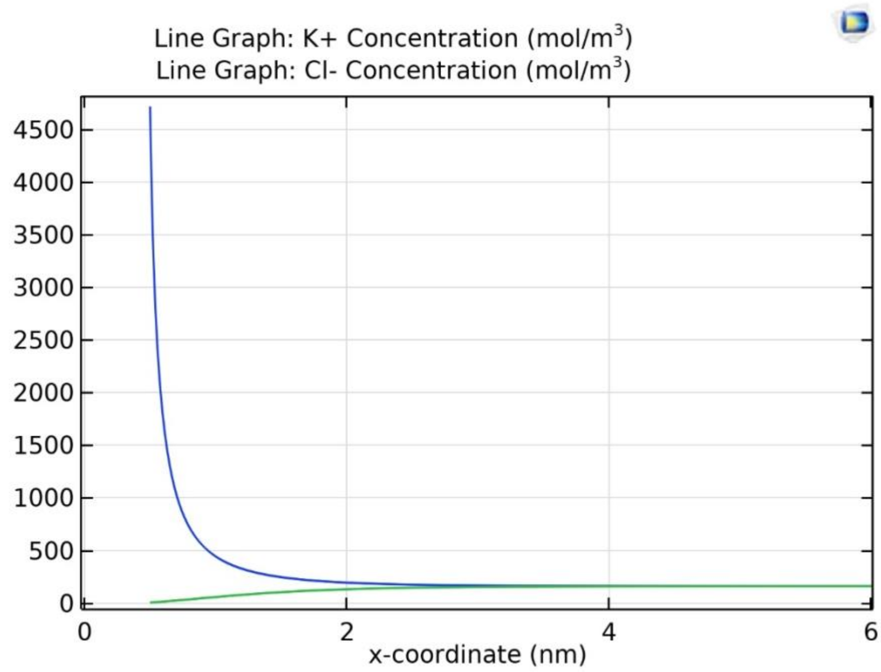

Supplementary Figure 1. Plots of the K^+ and Cl^- concentration around a CT with the Stern Layer excluded from the simulations ($d = 0$). The simulations are of the NLPB equation with a variable permittivity and the x-coordinate is the radial distance from the centre of the CT. When these same simulations are run with the Stern Layer included ($d = 0.33\ nm$) the maximum K+ concentration, occurring at the Stern Layer, is 1664 mM. For this simulation, the K+ concentration 0.83 nm away

from the centre of the CT is 655 mM. This highlights the importance of including the Stern Layer correction when calculating the potential and counterion concentrations around a CT.

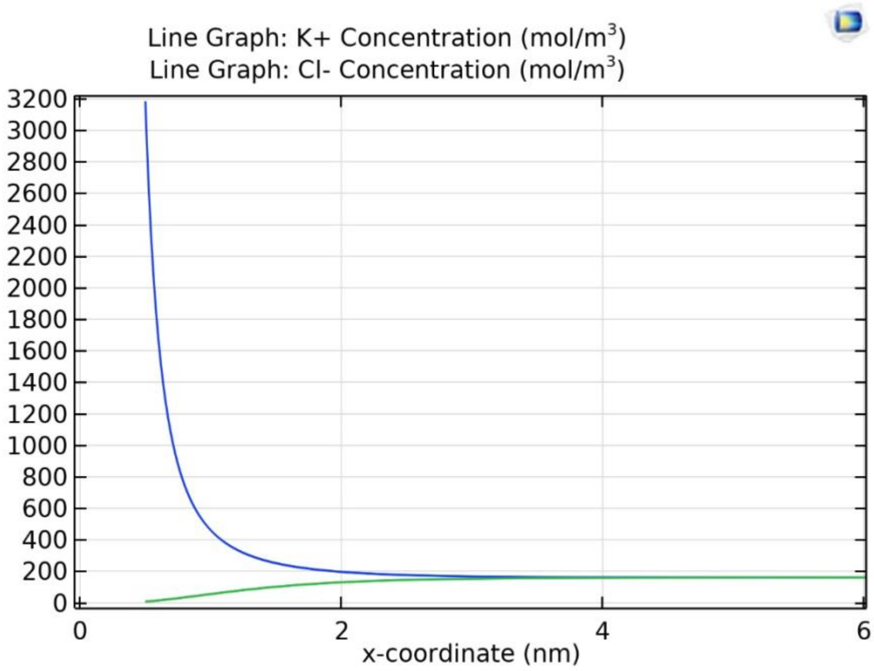

Supplementary Figure 2. Plots of the K^+ and Cl^- concentration around a CT with the Stern Layer excluded from the simulations ($d = 0$). The simulations are of the NLPB equation with a constant relative permittivity and the x-coordinate is the radial distance from the centre of the CT. When these same simulations are run with the Stern Layer included ($d = 0.33\ nm$) the maximum K+ concentration is 1503 mM and occurs at the Stern Layer (x = 0.83 nm). For the simulation shown here the concentration at this location is 685 mM. This highlights the importance of including the Stern Layer correction when calculating the potential and counterion concentrations around a CT.
